# WOMEN's Knowledge of Obstetric Danger signs in Ethiopia (WOMEN's KODE):a systematic review and meta-analysis

**DOI:** 10.1186/s13643-019-0979-7

**Published:** 2019-02-25

**Authors:** Ayele Geleto, Catherine Chojenta, Abdulbasit Musa, Deborah Loxton

**Affiliations:** 1 School of Public Health, College of Health and Medical Sciences, Haramaya University, Harar, Ethiopia; 2 Research Centre for Generational Health and Ageing, Faculty of Health and Medicine, School of Medicine and Public Health, the University of Newcastle, Newcastle, Australia; 3 School of Nursing and Midwifery, College of Health and Medical Sciences, Haramaya University, Harar, Ethiopia

**Keywords:** Women’s health, Pregnancy, Obstetric danger signs, Ethiopia

## Abstract

**Background:**

According to the 2015 World Health Organization report, globally, an estimated 10.7 million mothers died from 1990 to 2015 due to obstetric complications. This report showed that almost all global maternal deaths (99%) occurred in developing countries and two thirds of these deaths took place in sub-Saharan Africa where the majority of women lack knowledge about obstetric danger signs. In Ethiopia, in several research reports, it has been indicated that women have poor knowledge about obstetric danger signs. Although several studies have been conducted to assess women’s knowledge of obstetric danger signs, to date, no systematic review has been conducted in Ethiopia. Therefore, this review is aimed at synthesising the existing literature about women’s knowledge of obstetric danger signs.

**Methods:**

We systematically searched for articles from MEDLINE, Cumulative Index to Nursing and Allied Health Literature, Embase, Web of Science, Scopus, Google Scholar and Maternity and Infant Care databases. A combination of search terms including ‘knowledge’ or ‘awareness’ or ‘information’ and ‘pregnancy danger signs’ or ‘obstetric danger signs’ or ‘obstetric warning signs’ and ‘Ethiopia’ was used to locate appropriate articles. Two reviewers conducted article screening and data abstraction independently. Observational studies published in English and conducted in Ethiopia to date were assessed for quality using the adapted Newcastle Ottawa Scale for cross-sectional studies. The PRISMA checklist was used to present the findings of this systematic review.

**Results:**

From the 215 articles initially screened by abstracts and titles, 12 studies fulfilled the inclusion criteria. All the studies reported women’s knowledge of obstetric danger signs during pregnancy, ten articles reported on the level of knowledge during delivery and eight studies reported on the level of knowledge of danger signs during the postpartum period. The pooled random effect meta-analysis level of women’s knowledge about obstetric danger signs during pregnancy, delivery and postpartum was 48%, 43% and 32%, respectively. Maternal age, education, income, health service use, distance from facility and women’s autonomy were reported in several studies as determinants of women’s knowledge of obstetric danger signs.

**Conclusions:**

Women’s knowledge about obstetric danger signs in Ethiopia was very poor, which could hamper access to obstetric care when women encounter obstetric complications. Counselling services during antenatal care and community-based health information dissemination about obstetric danger signs should be strengthened.

**Systematic review registration:**

PROSPERO CRD42017077000

**Electronic supplementary material:**

The online version of this article (10.1186/s13643-019-0979-7) contains supplementary material, which is available to authorized users.

## Background

Pregnant women are at risk of facing complications arising during pregnancy, and 15% of all pregnant mothers develop a specific obstetric complication [1]. The main obstetric complications that women could encounter during pregnancy and childbirth include maternal haemorrhage, pregnancy-induced hypertension, maternal infections, prolonged/obstructed labour and complications of abortion [2–4]. An estimated 10.7 million mothers died from 1990 to 2015 due to obstetric complications [5]. Almost all of these deaths (99% of global maternal mortalities) occurred in developing countries, and 66% of these deaths occurred in sub-Saharan African countries [6].

The majority of obstetric complications can be prevented if its occurrence is recognized and women receive timely quality obstetric care [7, 8]. Globally, a noticeable reduction in the maternal mortality ratio (MMR) has occurred since 1990, primarily due to the provision of effective and quality emergency obstetric care (EmOC) [9]. However, in most sub-Saharan African countries, the MMR has remained stagnant over the last two decades and few countries showed encouraging improvements [5, 10]. The high maternal mortality in developing countries is attributed to women’s poor access to EmOC [11–13]. Ethiopia, with a current MMR of 412 per 100,000 live births, remains among one of the African countries with the highest maternal mortality [14].

In developing countries, several factors are reported to hamper access to EmOC. The most frequently reported deterrents include socio-economic and cultural factors [15–17], lack of knowledge about obstetric danger signs [18–20] and poor awareness of the availability of EmOC service [21–24]. Lack of knowledge about obstetric danger signs often results in delays in seeking timely obstetric care [25]. In Ethiopia, it has been indicated that only 6% of women who encountered obstetric complications were able to access EmOC [26], with the main reason being lack of knowledge about obstetric danger signs. In order to obtain timely obstetric care, women, families and the community at large need to know when to access healthcare. Knowledge of the major obstetric danger signs, including severe vaginal bleeding, oedema on the face, blurred vision, prolonged labour, convulsions, retained placenta, foul-smelling vaginal discharge, and high grade fever [27], can help to facilitate timely healthcare access.

In previous research in developing countries, it has been suggested women’s knowledge about obstetric danger signs determines their health-seeking behaviour. For instance, Kosum et al. [28] reported that women with poor knowledge of obstetric danger signs are less likely to attend a healthcare facility when they face obstetric emergencies. Similarly, Jammeh et al. [29] stated that an inability to identify danger signs during pregnancy by women was reported to result in delays in accessing obstetric care. Several researchers have indicated that women with poor knowledge of obstetric danger signs are less likely to have better birth preparedness and complication readiness, and as a result they usually delay seeking appropriate obstetric care [30–34].

At the global level, women’s knowledge about obstetric danger signs has been related to a number of factors. Women who experienced obstetric complications during the previous pregnancies [17, 35] are more knowledgeable about obstetric danger signs as compared to those who never experienced obstetric complications. Being exposed to health education [36–40] was also reported to improve women’s knowledge about obstetric danger signs. In numerous studies, it has been shown that multiparous [17, 39, 41, 42] and those women who visited a health facility for antenatal care (ANC) [17, 40, 43] are more likely to be aware of obstetric danger signs as compared to nulliparous and those who did not visit a health facility for ANC.

Maternal socio-economic factors are also reported in several studies as affecting women’s knowledge about obstetric danger signs. Women who are employed and work for paid jobs [36, 38, 41, 43, 44] are more knowledgeable about obstetric danger signs compared to their unemployed counterparts. The result of numerous studies showed that older women [15, 39, 42, 44–46] and educated mothers [16, 17, 36, 38–46] are more knowledgeable about obstetric danger signs than their younger and uneducated counterparts. Morhason-Bello and colleagues, in their study of Nigeria, showed that the Islamic religion [47] was associated with better knowledge of obstetric danger signs. Autonomous women who are able to make important decisions by themselves [48–50] and those who have higher household income [16, 43] are reported to have better knowledge of obstetric danger signs.

In Ethiopia, similar socio-demographic determinants of women’s knowledge about obstetric danger signs have been reported. In the findings of several studies, it has been shown that educated mothers [18, 19, 31, 51–55], older women [54, 56–58] and employed women [52, 57, 59] have relatively better knowledge than the uneducated, younger and unemployed mothers. Better knowledge of obstetric danger signs was also reported among women who have an educated partner [57], have higher household income [18] and are urban dwellers [53]. Women who previously gave birth at a health facility [18, 52, 54, 55], those with higher number of parities [18, 52, 57] and those who visited a health facility for ANC services in previous pregnancies [18, 19, 31, 52, 55, 58, 60] were reported to have comparatively better knowledge about obstetric danger signs.

In Ethiopia, there were several studies about women’s knowledge of obstetric danger signs and associated factors. Majority of the available studies are cross-sectional in design and conducted in limited areas; hence, we are unable to indicate more accurately women’s knowledge at the national level. Further, the available data have not been systematically combined to generate a precise understanding of the levels of women’s knowledge about obstetric danger signs. A systematic review would help policy formulators and health managers to make evidence-based decisions that have taken into account all the available information, as well as providing an indication as to the quality of the results. Therefore, this systematic review is designed to identify the level of women’s knowledge about obstetric danger signs to present accurate information that could be used in policy formulation and practice evidence-based decision-making. The findings of this review will be used in planning maternal health interventions to make-evidence based decisions by health service managers and policy formulators in Ethiopia.

## Methods

**Fig. 1:**
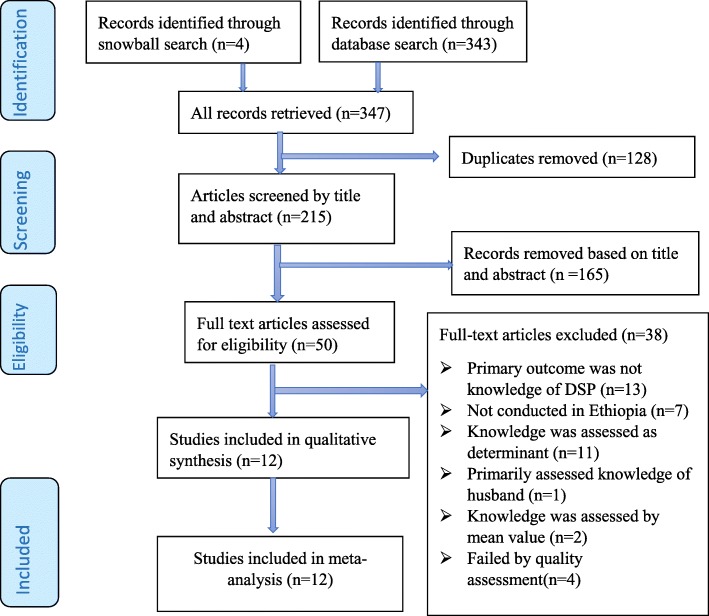
PRISMA flow diagram showing the selection of the included articles

The study protocol of this systematic review and meta-analysis was registered in the International Prospective Register of Systematic Reviews (PROSPERO 2017); ID CRD42017077000. The findings of this review were reported following the Preferred Reporting Items for Systematic Review and Meta-Analysis (PRISMA) checklist [61] [Fig. 1]. We also employed the Meta-analysis Of Observational Studies in Epidemiology (MOOSE) guidelines to conduct the meta-analysis and to report the results [62].

### Data source and search strategy

Published papers were searched online from MEDLINE, CINAHL, Embase, Web of Science, Scopus, Google Scholar and Maternity and Infant Care databases using appropriate search terms. The comprehensive search strategy and terms used to locate appropriate articles were developed in consultation with and expert librarian using appropriate Boolean operators. The MEDLINE database search strategy and terms are presented in [Additional file 1]. The core search terms and phrases were ‘knowledge’ or **‘**awareness’ or ‘information’ and ‘pregnancy danger signs’ or ‘obstetric danger signs’ or ‘obstetric complications’ or ‘warning signs during pregnancy’ or ‘obstetric warning signs’ or ‘gestational danger signs’ and ‘Ethiopia’. A combination of terms that could describe obstetric danger signs was also used to locate appropriate articles. These terms and phrases included ‘severe vaginal bleeding’, ‘blurred vision’, ‘swelling of face’, ‘convulsion’, ‘prolonged labour’, ‘retained placenta’, ‘foul smelling vaginal discharge’ and ‘grade fever’. We also tried to manually search for grey literature to include in to the review, however, no grey literature specific to the topic of interest was found.

### Inclusion and exclusion criteria

This review was designed to include studies conducted with cross-sectional, cohort and case-control study designs. Observational studies conducted in Ethiopia with both quantitative and qualitative methods were considered for inclusion. Although the search was not limited to specific language, only articles published in the English language were returned during the search process. All articles reported the level of women’s knowledge about obstetric danger signs were considered for the review. Articles were included irrespective of their publication year. Articles that assessed knowledge of women about obstetric danger signs based on the percentage of women who spontaneously mentioned at least two [63] and at least three [59] primary obstetric danger signs were included, and the analysis for both categories (knowledge of at least two or at least three danger signs) was conducted separately. Studies that reported women’s knowledge of obstetric danger signs during pregnancy, childbirth and postpartum periods were included, and subgroup analysis was conducted for each stage. Commentaries, studies that failed to fulfil the quality criteria (see below), anonymous reports, letters and editorials were excluded from this review.

Danger signs during pregnancy-included severe vaginal bleeding, swelling of face/hand, blurred vision and convulsions. Danger signs during labour and childbirth included severe vaginal bleeding, prolonged labour (> 12 h), retained placenta (> 1 h) and convulsion. The key danger signs during the postpartum period included severe vaginal bleeding, foul smelling vaginal discharge, severe lower abdominal pain and high grade fever [27].

### Screening of the articles

Initially, the articles obtained from the selected databases were exported to EndNote x8.1 version library and exact duplicates were removed. Then, the EndNote library was shared between two reviewers who independently screened articles by title and abstract. After finalization of abstract review by the two reviewers, Cohen’s kappa coefficient was performed to judge for agreement between the reviewers. Acceptable agreement between the reviewers was concluded as substantial agreement (> 0.60) [64] from the Cohen’s kappa coefficient obtained. Any disagreements between the reviewers were solved through discussion. After reaching a consensus, the full-text review was performed by the two reviewers independently.

### Data extraction

Two reviewers independently extracted the data from the full text of the retained articles. The data were extracted by using an adapted Johanna Briggs Institute (JBI) data abstraction format [65]. Study characteristics including name of the first author and publication year, region where the study was conducted, objective of the study, study design, study population, sampling method, sample size, data collection procedure, level of women’s knowledge and factors affecting women’s knowledge were abstracted from all included articles [Additional file 2].

### Quality and risk of bias assessment

The quality of the retained articles was appraised independently by the two reviewers using an adapted version of Newcastle–Ottawa Scale (NOS) for cross-sectional studies [66], since all of the articles that fulfilled the inclusion criteria were conducted with cross-sectional designs. The NOS was reported to have a good inter-rater reliability and validity [67] and was applied in observational study [68]. The tool gives a maximum score of nine with three categories of criteria. A maximum of four stars allotted for ‘sample selection’; a maximum of two stars allotted for ‘comparability’; and a maximum of three stars allotted for the ‘outcome’. Finally, only those articles with good quality, that is, those articles which obtained seven or more stars, were included in the final review and analysis. Quality scores for each article are presented in [Additional file 3].

### Outcome measure and data synthesis

The primary outcome variable of this review was the level of women’s knowledge about obstetric danger signs and was measured based on the proportion of women who spontaneously mentioned the key obstetric danger signs. The level of women’s knowledge about obstetric danger signs reported in different studies was presented by pooling the level of women’s knowledge reported in the included articles. To take the study-specific true effects across the included studies into consideration, the random effect meta-analysis model was employed. A random effects model for the reported proportion was used to present the pooled knowledge of women about obstetric danger signs that occurred at different stages of pregnancy and child birth. In this review, different factors that are reported to affect the level of women’s knowledge about obstetric danger signs were identified. These factors were presented using a narrative synthesis through generation of different themes.

### Subgroup analysis

Subgroup analysis was carried out based on the region where the study was conducted, as defined by the Federal Democratic Republic of Ethiopia, and the place where the study was conducted, whether facility based or community based. Subgroup analysis was also done for articles which assessed knowledge of women based on the number of key obstetric danger signs women spontaneously mentioned.

## Results

The systematic search of seven selected databases yielded 343 articles. Four additional studies were identified from other sources such as searching the reference lists of relevant articles and communicating with the authors of the included studies for further information. There were 215 studies remaining after duplicates were removed and further screened by abstracts and titles. After screening titles and abstract, 50 relevant articles were retained for the full-text review. There were 38 papers that did not meet the inclusion criteria, leaving 12 papers [51, 54, 56–60, 63, 69–72] for the final systematic review and meta-analysis (Fig. 1). Though the review was designed to include cohort studies, cross-sectional and case-control studies, all the identified studies were conducted with cross-sectional study design. Despite our plan to include quantitative and qualitative studies, no qualitative studies were returned from the database search. Irrespective of the unlimited study period, only articles published in 2010 and later were found to satisfy the inclusion criteria.

Table 1 contains the basic characteristics of the included papers according to the level of women’s knowledge about obstetric danger signs. The retained 12 studies [51, 54, 56–60, 63, 69–72] were conducted in different regions of Ethiopia. The majority of the studies were conducted in three regions of the country. Three of the studies (25%) were conducted in the Amhara region [51, 60, 71], three studies (25%) in the Southern Nations, Nationalities and People region [56, 58, 59] and three studies (25%) in the Oromia region [54, 69, 70]. Two studies (17%) were conducted in the Tigrai region [57, 72] while the remaining study (8%) was conducted in the Ethiopian Somali region [63]. Half of these studies were conducted in health facilities [54, 57, 59, 60, 70, 71] and the remaining half were community-based studies [51, 56, 58, 63, 69, 72].

**Table 1:** Characteristics of the studies included in this systematic review

Authors and year	Region	Recruitment year	Setting	Spontaneous response	Study design	Respondents	Women’s knowledge of obstetric danger signs during			Quality score
							Pregnancy *n* (%)	Childbirth *n* (%)	Postpartum *n* (%)	
Abiyot T et al. (2014) [57]	Tigray	2013	Facility	At least 2	Cross sectional	359	296 (82.5)	–	–	8star
Bililign N. et al. (2017) [51]	Amhara	2016	Community	At least 3	Cross sectional	493	230 (46.7)	137 (27.8)	130 (26.4)	9star
Bogale D. et al. (2015) [69]	Oromia	2013	Community	At least 3	Cross sectional	562	179 (31.9)	152 (27)	124 (22.1)	8star
Damme T. G. et al. (2016) [70]	Oromia	2015	Facility	At least 3	Cross sectional	198	152 (76.8)	154 (77.8)	129 (65.5)	8star
Endalemaw et al. (2014) [71]	Amhara	2012	Facility	At least 3	Cross sectional	385	181(47)	176(45.7)	–	7star
Hailu D. et al. (2014) [72]	Tigray	2013	Community	At least 2	Cross sectional	485	285 (58.8)	299 (61.6)	–	8star
Hailu M. et al. (2010) [56]	SNNPR	2007	Community	At least 2	Cross sectional	743	226 (30.4)	305 (41.3)	279 (37.7)	8star
Hibstu D. T. et al. (2017) [59]	SNNPR	2016	Facility	At least 3	Cross sectional	342	168 (49.1)	181 (52.9)	153 (44.7)	9star
Maseresha N et al. (2016) [63]	Somali	2014	Community	At least 2	Cross sectional	632	201 (31.8)	161 (25.5)	121(19.1)	9star
Workineh Y. et al. (2014) [58]	SNNPR	2014	Community	At least 2	Cross sectional	390	184 (47.2)	193(49.5)	285(73)	8star
Solomon A. et al. (2015) [60]	Amhara	2014	Facility	At least 2	Cross sectional	355	137(38.6)	–	–	8star
Tsegaye D. et al. (2017) [54]	Oromia	2015	Facility	At least 2	Cross sectional	831	309 (37.3)	194 (23.3)	30 (3.6)	8star

*SNNPR* Southern Nations, Nationalities and People Region

From the retained 12 studies, eight reported the level of women’s knowledge about obstetric danger signs throughout the perinatal period: during pregnancy, labour and immediate postpartum periods [51, 54, 56, 58, 59, 63, 69, 70]. Ten of the studies reported the primary outcome variable only at two stages: during pregnancy and childbirth [51, 54, 56, 58, 59, 63, 69–72], while all of the studies reported the level of women’s knowledge about obstetric danger signs during pregnancy (Table 1).

### Pooled meta-analysis in different outcome categories

#### Level of women’s knowledge during pregnancy

**Fig. 2:**
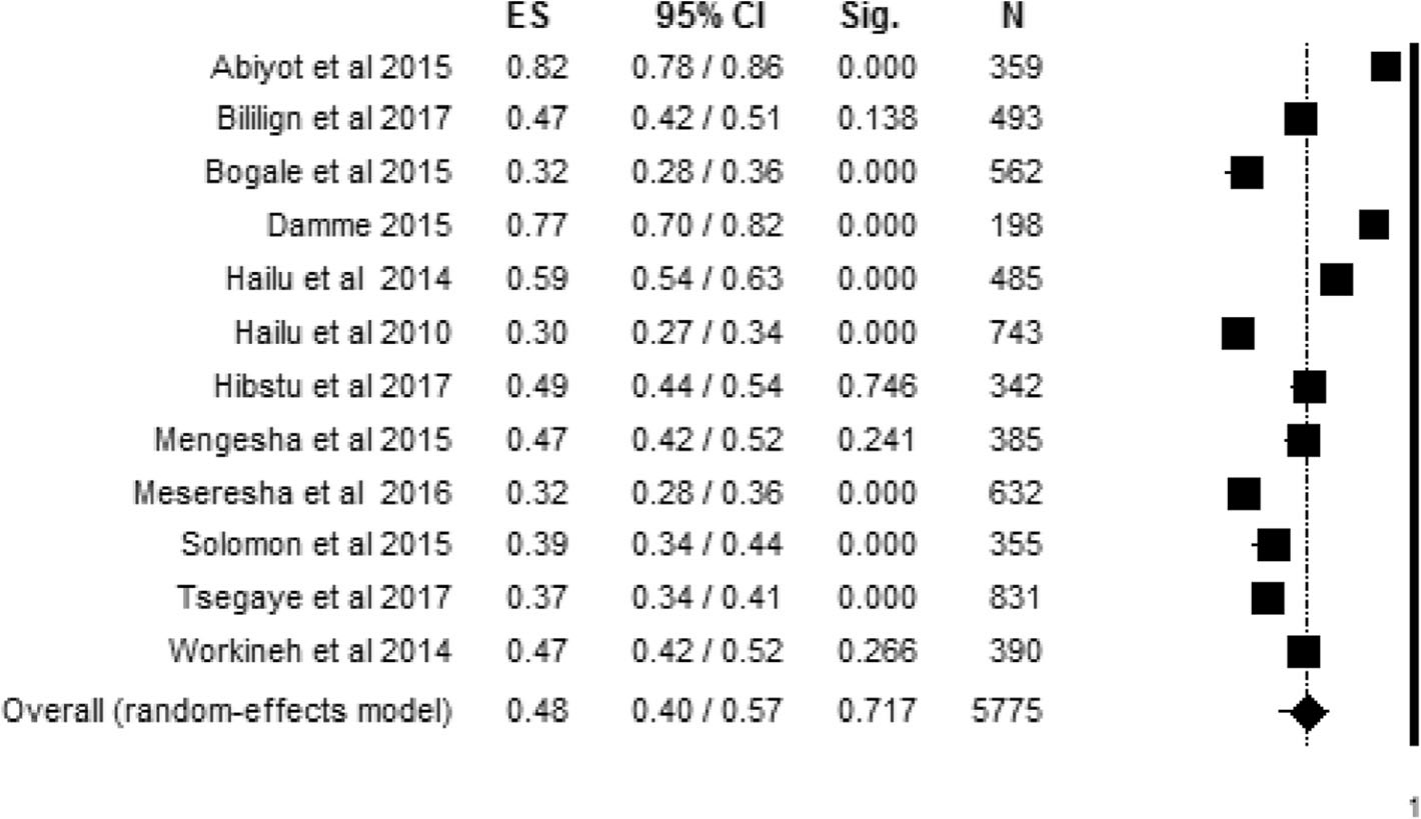
Random effect meta-analysis level of women’s knowledge about obstetric danger signs during pregnancy in Ethiopia

Level of women’s knowledge about obstetric danger signs during pregnancy with 12 individual study populations ranged between 30% and 82%, with an overall summarized random effect meta-analysis level of 48% [95% CI; (40%, 57%), *I*^2^ = 97.45, *p* < 0.001**,** *n* = 5775] (Fig. 2).

#### Level of women’s knowledge during delivery

**Fig. 3:**
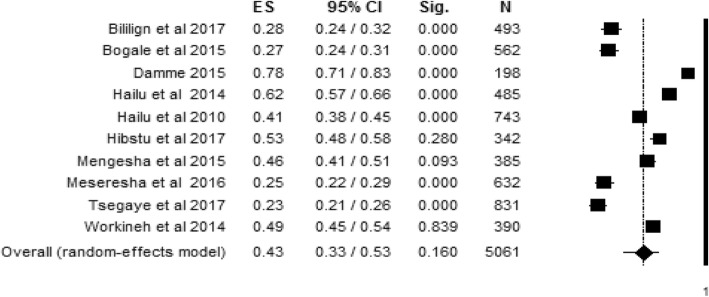
Random effect meta-analysis level of women’s knowledge about obstetric danger signs during delivery in Ethiopia

The point prevalence of women’s knowledge about obstetric danger signs during delivery with the ten individual study populations ranged between 23% and 78%, with an overall summarized random effect meta-analysis prevalence of 43% [95% CI; (33%, 53%), *I*^2^ = 97.94, *p* < 0.001, *n* = 5061] (Fig. 3).

#### Level of women’s knowledge during postpartum

**Fig. 4:**
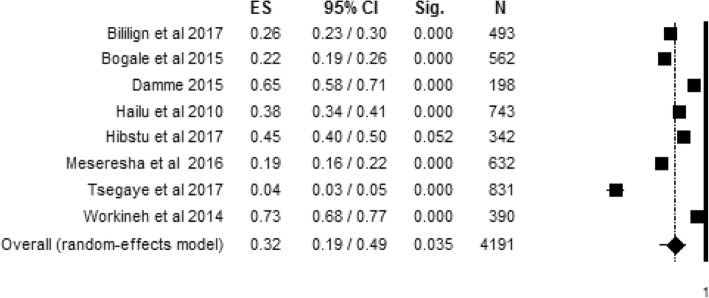
Random effect meta-analysis level of women’s knowledge about obstetric danger signs during postpartum in Ethiopia

The level of women’s knowledge about obstetric danger signs that occurred after childbirth with eight individual study populations was found to range between 4% and 73%, with an overall summarized random effect meta-analysis knowledge of 32% [95% CI; (19%, 49%), *I*^2^ = 98.86, *p* < 0.001, *n* = 4191] (Fig. 4).

**Fig. 5:**
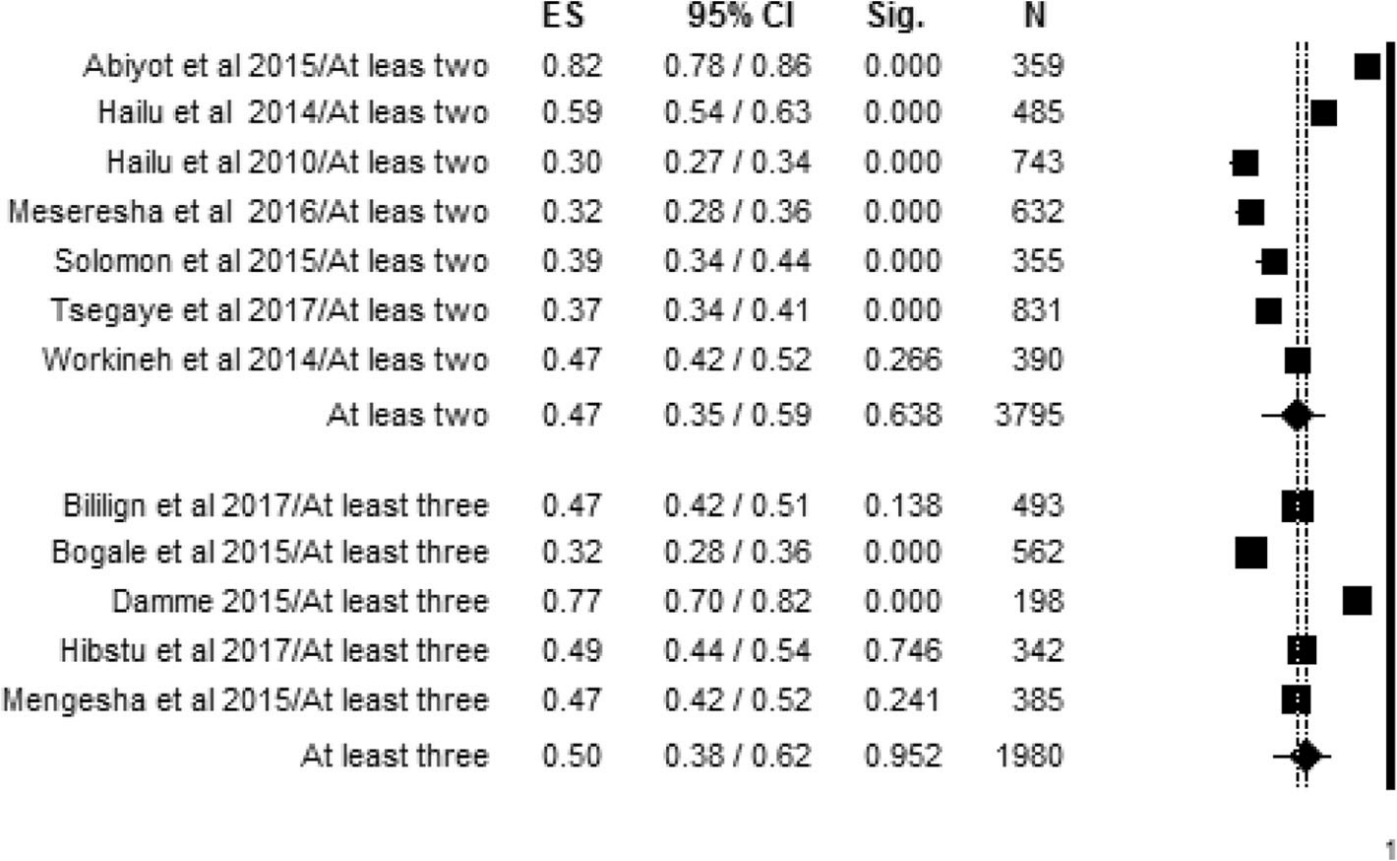
Level of women’s knowledge about obstetric signs during pregnancy based on the number of danger signs women mentioned

Subgroup analysis of women’s knowledge about obstetric danger signs during pregnancy was conducted based on the number of spontaneous responses given by women. Seven articles assessed women’s knowledge based on at least two spontaneous responses given by women [54, 56–58, 60, 63, 72] and the remaining five articles assessed based on at least three spontaneous responses [51, 59, 63, 69, 70]. Accordingly, the level of women’s knowledge about obstetric danger signs during pregnancy with seven individual study populations assessed with at least two spontaneous responses was found to range between 30% and 82%, with an overall summarized random effect meta-analysis knowledge of 47% [95% CI; (33%, 59%), *I*^2^ = 98.10, *p* < 0.001, *n* = 3795]. Women’s knowledge about obstetric danger signs during pregnancy with five individual study populations assessed with at least three responses was found to range between 32% and 77%, with an overall summarized random effect meta-analysis knowledge of 50% [95% CI; (38%, 62%), *I*^2^ = 96.37, *p* < 0.001, *n* = 1980] (Fig. 5).

**Fig. 6:**
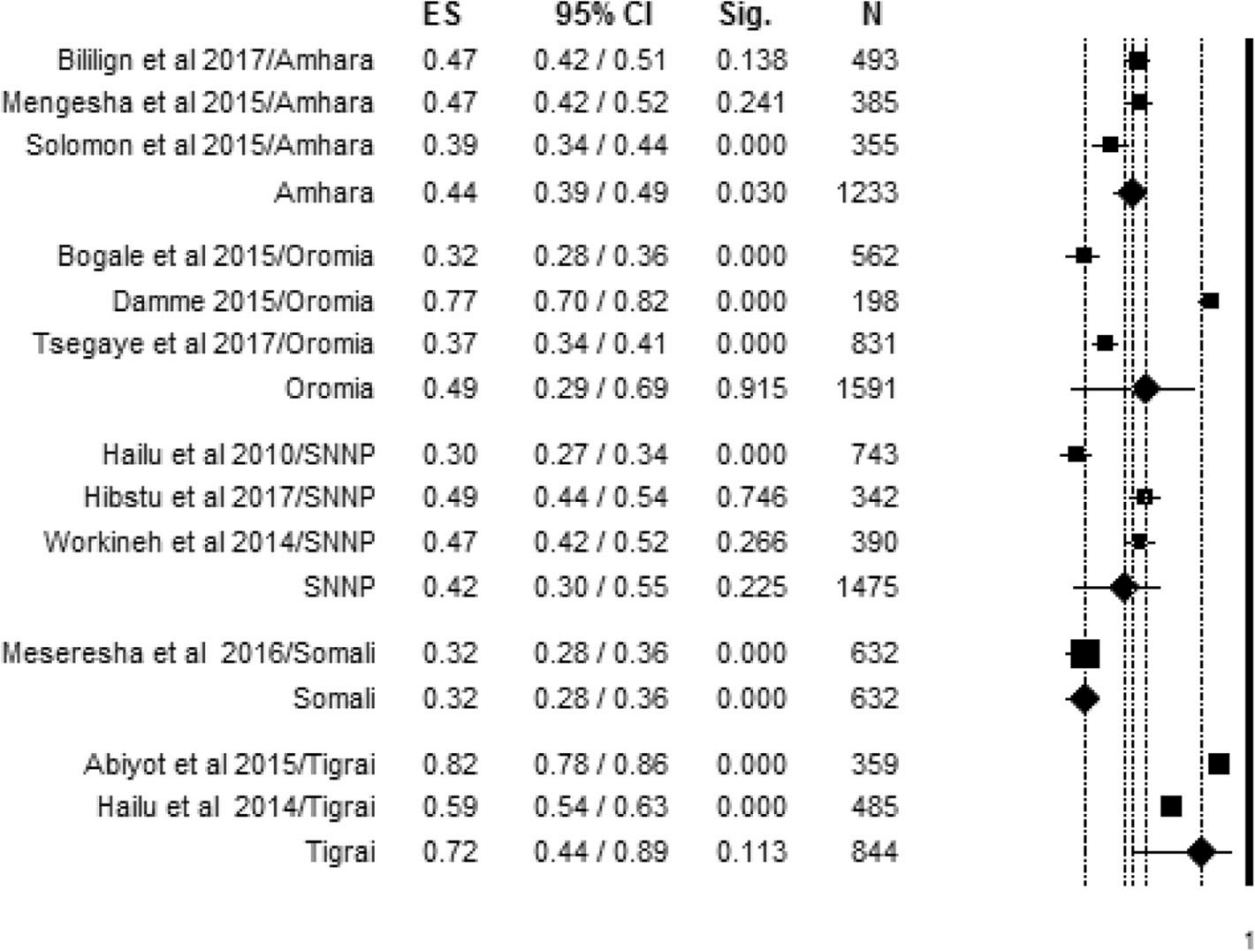
Regional variation of level of women’s knowledge about obstetric danger signs during pregnancy

Regional variation in knowledge of women about obstetric danger signs during pregnancy was also observed in this review. The 12 included articles were conducted in five different regions of the country, and level of women’s knowledge about obstetric danger signs was found to range between 32% in the Ethiopian Somali region with single study and 72% in random effect pooled meta-analysis in the Tigrai region. The overall summarized random effect meta-analysis level of women’s knowledge about obstetric danger signs was 44% [95% CI; (39%, 49%), *I*^2^ = 71.02, *p* < 0.032, *n* = 1233] in the Amhara region. In the Oromia region and Southern Nations, Nationalities. and Peoples’ (SNNP) region, it was found to be 49% [95% CI; (29%, 69%), *I*^2^ = 98.15, *p* < 0.001, *n* = 1591] and 42% [95% CI; (30%, 55%), *I*^2^ = 95.83, *p* < 0.001, *n* = 1475], respectively (Fig. 6).

**Fig. 7:**
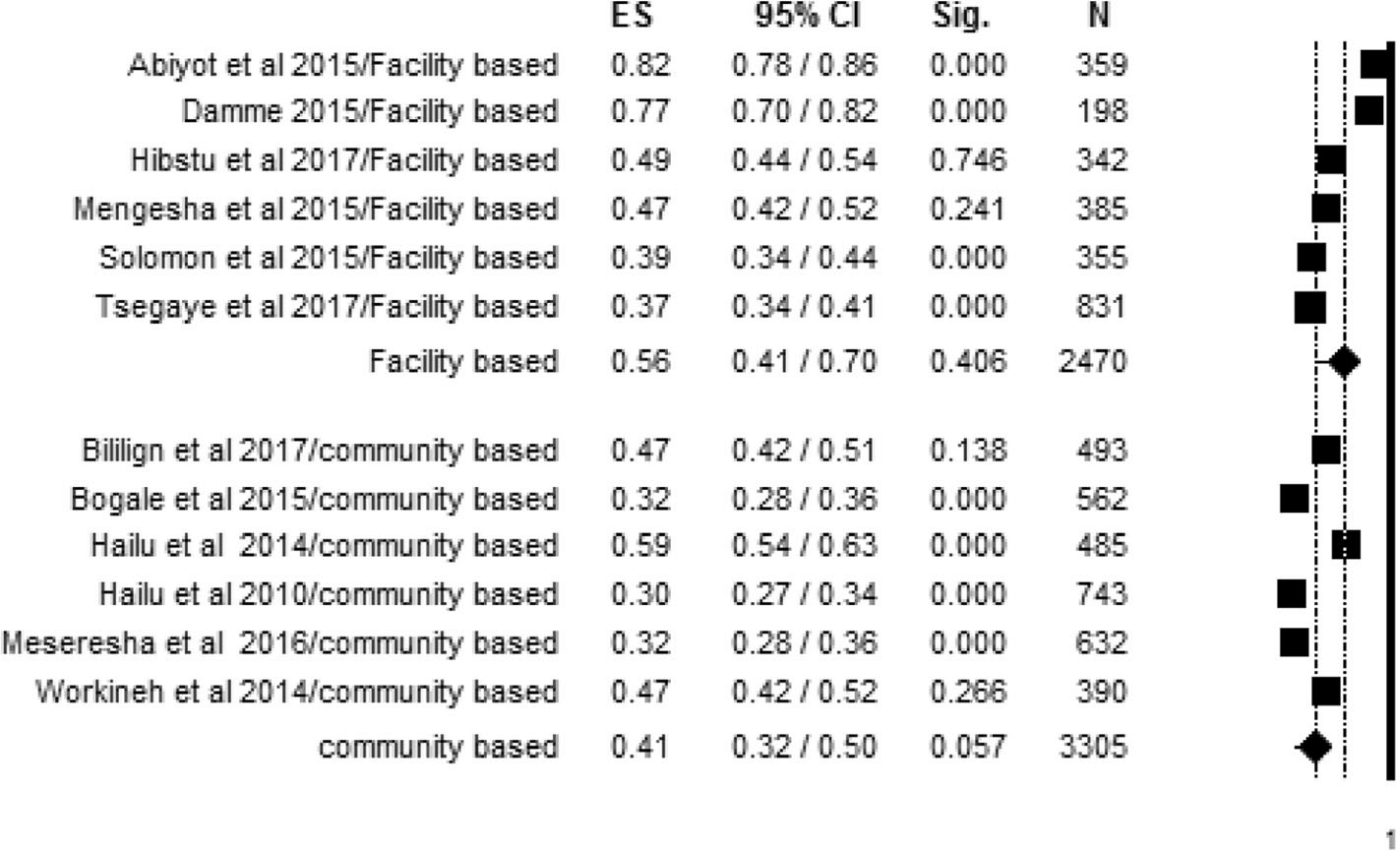
Subgroup analysis for level of knowledge about obstetric danger signs during pregnancy among studies conducted at facility and community

Variation in the knowledge of obstetric danger signs during pregnancy was also observed between findings of studies conducted in a health facility and community-based studies. Accordingly, the overall summarized random effect meta-analysis level of women’s knowledge about obstetric danger signs was found to be 56% [95% CI; (41%, 70%), *I*^2^ = 97.97, *p* < 0.001, *n* = 2470] for studies conducted in health facilities and 41% [95% CI = (32%, 50%), *I*^2^ = 96.58, *p* < 0.001, *n* = 3305] for community-based studies (Fig. 7).

### Factors affecting knowledge of women about obstetric danger signs

Different factors were reported in several studies as determinants of women’s knowledge about obstetric danger signs. While compiling and synthesizing these factors, four themes emerged from the factors that are reported in different studies. These themes include socio-demographic factors, reproductive history of the women, health service use and other miscellaneous factors (Table 2).

**Table 2:** Factors affecting women’s knowledge of obstetric danger signs in Ethiopia

Thematic area	Factors associated with better knowledge of women	Studies
Socio-demographic factors	Younger women	[58, 59]
	Older women	[54, 57, 63]
	Educated mothers	[51, 54, 56, 58–60, 70–72]
	Higher household income	[58, 69]
	Having educated partners	[57, 59, 69]
	Employed mothers	[51, 57, 69–71]
	Having employed partners	[59]
	Married women	[56]
	Urban residence	[56, 59, 60, 63, 69]
Reproductive history	Higher gravidity and parity	[56, 57, 63]
	Previous prolonged labour	[73]
Health service use	Having ANC visit	[51, 60, 63, 69, 71]
	Previously gave birth at health facility	[51, 54, 72]
	Satisfaction with the service	[54]
	Distance < 30 min from facility	[59, 69]
Miscellaneous factors	Exposure to media	[70, 72]
	Autonomous women	[54, 58]

### Socio-demographic factors

Socio-demographic factors were reported in the majority of the studies as determinant factors of women’s knowledge about obstetric danger signs. In some of the studies, it was reported that younger women [58, 59] have better knowledge while several studies indicated older women [54, 57, 63] were relatively knowledgeable about obstetric danger signs. In the majority of the studies, it was shown that mothers who attended any formal education [51, 54, 56, 58–60, 70–72] were more knowledgeable than women who attended no formal education. Those women who have an educated husband [57, 59, 69] were also reported to be more knowledgeable about obstetric danger signs. Employed mothers and those who worked for paid jobs were reported in different studies [51, 57, 69–71] to have better knowledge of obstetric danger signs than unemployed women. Knowledge of obstetric danger signs was also found to be better among women with an employed partner [59]. It was also reported that women who have higher household income [58, 69] and married women [56] were more likely to have a higher level of knowledge about obstetric danger signs. Several researchers showed that urban dwellers have better knowledge of obstetric danger signs compared to their rural counterparts [56, 59, 60, 63, 69] (Table 2).

### Women’s reproductive history

The reproductive history of women emerged as a determinant factor of the level of women’s knowledge about obstetric danger signs. In this theme, a higher number of pregnancies and childbirth were reported in several studies [56, 57, 63] to be associated with better knowledge of women about obstetric danger signs. In one study, it was shown that women who experienced prolonged labour in their previous pregnancies were more likely to know about obstetric danger signs than those who experienced no obstetric complications [73].

### Health service use

Attending health facilities for utilization of maternal health service were reported in a number of studies to affect women’s knowledge about obstetric danger signs. Women who visited a facility for ANC services [51, 52, 60, 63, 69, 71], those who previously gave birth at a health facility [51, 52, 54, 72], and those who were satisfied with the service [54] were reported to have a better awareness of obstetric danger signs, compared with those who had not previously accessed the services. Knowledge of obstetric danger signs was also reported to be lower among women who travelled for more than 30 min to health facilities for health service utilization [59, 69].

### Other miscellaneous factors

A number of miscellaneous factors other than socio-demographic, health service utilization and reproductive history were reported in different articles to affect women’s knowledge of obstetric danger signs. Women who have the means of exposure to media [70, 72] and autonomous women who can decide to use health service [54, 58] were reported to have better knowledge of obstetric danger signs (Table 2).

## Discussion

The pooled random effect meta-analysis showed that knowledge of Ethiopian women about obstetric danger signs was very low. This low level of knowledge may be attributed to the lack of exposure to health information during pregnancy since ANC utilization, skilled delivery and PNC utilization are very low in Ethiopia [14, 74]. In studies conducted in India [40] and Tanzania [36], it was revealed that the lack of exposure to formal health counselling was found to be significantly associated with poor knowledge about obstetric danger signs among women. Similarly, Duysburgh and colleagues found that counselling during pregnancy can significantly improve women’s knowledge of obstetric danger signs [42].

This review presented a comprehensively synthesised report of the estimated level of women’s knowledge about obstetric danger signs. In general, 12 observational studies conducted in Ethiopia, which reported on 5775 women in the reproductive age group, were included. The random effect pooled meta-analysis level of women’s knowledge about obstetric danger signs during pregnancy was 48% (40%, 57%). The level of women’s knowledge about obstetric danger signs was found to be 43% (33%, 53%) during delivery/childbirth and 32% (19%, 49%) during immediate postpartum respectively. In this review, socio-demographic factors, reproductive history of the women and maternal health service utilization were reported in a number of studies as determinant factors of the level of women’s knowledge about obstetric danger signs.

An important regional variation of women’s knowledge about obstetric danger signs during pregnancy emerged during subgroup analysis. Articles were included from five different regions in Ethiopia, and the level of women’s knowledge about obstetric danger signs during pregnancy was lowest (32%) in the Ethiopian Somali region, eastern Ethiopia. The highest level of women’s knowledge about obstetric danger signs (72%) was reported in the Tigrai region, northern Ethiopia. The overall summarized random effect meta-analysis level of knowledge about pregnancy danger signs in the SNNP, Amhara and Oromia regions were found to be 42%, 44% and 49%, respectively. This variation could be attributed to the disparities in maternal health service utilization at different regions of Ethiopia. Yusuf et al. [75], for example, found that ANC use is highest in the Tigrai region and lowest in the Ethiopian Somali region. Similarly, Bobo et al. [76] reported maternal health service utilization was highest in the Tigrai region and lowest in the Ethiopian Somali region. The probability of having a counselling service on obstetric danger signs among women living in a low maternal health utilization region can be very low.

Disparities in the level of women’s knowledge about obstetric danger signs were also observed between studies conducted in health facilities and community-based studies. After pooling and conducting the random effect meta-analysis model, a higher level of women’s knowledge about obstetric danger signs during pregnancy was found in studies conducted in health facilities, compared to community-based studies (56% vs 41%). In facility-based studies, the study participants were those females who came to the health facility to use maternal health services. Therefore, they may have more information about healthcare and well-being than the participants of a community-based study so that they responded to more questions than the participants of community-based studies [37, 52].

Maternal education level was the most frequently reported determinant of women’s knowledge about obstetric danger signs. Mothers who attended some level of education were reported to have a higher level of knowledge about obstetric danger signs compared to uneducated mothers. In studies conducted in Ethiopia [18, 19, 31, 52, 55] and other African countries [15–17, 39], it was reported that women’s educational status was a determinant of women’s knowledge about obstetric danger signs. Similarly, a lower level of women’s knowledge of obstetric danger signs was reported among uneducated women in Asian countries [40, 46]. This difference may be attributed to health information exposure among educated women that could be obtained from school. Educated women usually have less difficulty understanding the information received from counselling during ANC visits as compared to their uneducated counterparts [42]. Furthermore, educated mothers can read and understand health messages from communication channels, including printed materials [77].

Maternal age was found to be another determinant factor of women’s knowledge about obstetric dangers signs. In the majority of the articles, it was reported that older women have better knowledge of obstetric danger signs compared to younger women. It was frequently reported that older African women have a higher level of knowledge about obstetric danger [39, 44, 45]. A higher level of knowledge about obstetric danger signs among mothers at an advanced age may be attributed to their own prior experiences of childbirth. These experiences helped women to be aware of important information, especially among those who have experienced obstetric complications during previous pregnancies [42]. Therefore, health education during ANC for younger mothers who lack the experience of child birth could enhance knowledge about obstetric danger signs. Women living in rural areas usually have lower educational status [18, 19, 31, 52, 55], so they often have poor awareness about health related danger signs [15, 16].

Maternal employment status and household income were frequently reported to play a major role in determining women’s knowledge about obstetric danger signs. Women’s employment usually improves household income and satisfies the financial needs of women, hence they can have access to health services, from which they obtain health-related information. Improved household financial need also helps the family to have access to communication materials, including a television and radio, that exposes them to health information [17, 38, 39]. Furthermore, employed women will have more opportunity to contact other women than unemployed women, which allows them to share experiences and gain more information about obstetric danger signs [38]. Mothers with a higher income rarely face cost barriers to seek medical care from where they learn more about obstetric danger signs [43].

Women’s reproductive experience was another important factor reported in various studies to affect the level of knowledge about obstetric danger signs. In the findings of different articles included in this review, it was indicated that women with multiple pregnancy and multiparous women have higher knowledge about obstetric danger signs. The level of knowledge about obstetric danger signs was also found to be higher among women who have experienced previous obstetric complications. Better awareness about obstetric danger signs among multigravida and multiparous women could be attributed to their own experiences of childbirth, which is an important source of their information [17, 18, 41, 42, 52], especially among those women who previously experienced obstetric complications [17, 39, 53].

Previous utilization of maternal health services was found to be another determinant factor that affected the level of women’s knowledge about obstetric dangers signs. This included utilization of ANC service during the previous pregnancies, giving birth previously at a health facility and level of satisfaction with the service. The positive effects of ANC utilization on knowledge of obstetric danger signs [17–19, 31, 39, 52, 55] may arise from counselling during the ANC service [36, 38–40]. Counselling during an ANC visit provides an excellent opportunity for women to obtain information, education and communication on pregnancy-related danger signs. Women who previously gave birth at a health facility were reported to have better knowledge of obstetric danger signs. This could be the result of the counselling service women receive from the health care provider during labour, delivery and postpartum, which may increase their knowledge of obstetric danger signs [18, 39, 43, 52, 55]. A longer walking distance to reach the nearest heath facility diminished the likelihood of mothers using health service [53], hence affecting women’s exposure to health information and counselling [36, 38, 39].

Finally, in this systematic review, a number of miscellaneous factors affecting knowledge of women about obstetric danger signs were reported in different articles. These factors include having exposure to media and women’s autonomy in decision making to use maternal health services. It is generally agreed that autonomous women often make their own decision to use maternal health services [48–50, 78] which creates a golden opportunity for them to be exposed to information about obstetric danger signs.

The limitations of this systematic review need to be acknowledged. First, the study method used in all the included articles was cross-sectional design. Therefore, this review shows the level of women’s knowledge only at a single point in time, and it is impossible to infer causal relationships among variables. There was no standardized method of measuring knowledge about obstetric danger signs and hence researchers have used their measuring method. Therefore, the result of this analysis was presented for articles which assessed the knowledge of women using ‘at least two spontaneous responses’ and ‘at least three spontaneous responses’ making it difficult to pool the level of knowledge together. In a number of articles, it was reported that women’s knowledge of obstetric danger signs in Ethiopia was excluded due to not fulfilling the inclusion criteria. The search for articles in this review was limited to databases of published literature and lacks inclusion of grey literature including government reports; hence, the result may not represent all regions of the country. Furthermore, there was no nationally representative study, making our finding difficult to compare with national findings.

This systematic review also had strengths. We used broader inclusion criteria to include studies conducted both at health facilities and in the community to capture a wider range of women’s knowledge about obstetric danger signs. Seven online databases were searched so as not to miss published studies that were conducted in Ethiopia. Furthermore, during the selection of articles, the PRISMA guideline was strictly followed and the articles were closely assessed for their quality.

## Conclusion

The pooled summarized random effect meta-analysis in this review revealed that less than half of women are knowledgeable about obstetric danger signs. The level of women’s knowledge about obstetric danger signs reduced over the perinatal period and was higher during pregnancy and lower during postpartum. Regional variation of women’s knowledge was observed in that women with the highest knowledge were reported in the Tigrai region and the lowest level of knowledge was reported in the Ethiopian Somali region. From this level of women’s knowledge, it can be concluded that only less than half of women have knowledge about obstetric danger signs. Health-seeking behaviour for the majority of women, when they face obstetric emergency, can be hampered by their lack of knowledge about obstetric danger signs. In this systematic review, socio-demographic factors, women’s reproductive history and health service utilization were found to affect the level of women’s knowledge about obstetric danger signs.

Therefore, to improve the health seeking behaviour of women when obstetric emergencies are faced, it is necessary to improve the awareness of the community, families and women about obstetric danger signs through factor-specific interventions. Counselling services about obstetric danger signs during ANC visits should be strengthened. Community-based health information enlightenment of groups of women [16] and use of electronic media to disseminate health information [79] could help women and the community at large to have a better awareness of obstetric danger signs. This may help the community to recognize any obstetric danger signs in a more timely fashion, and refer women to health facility so that they can receive appropriate lifesaving emergency obstetric care. Furthermore, national research should be conducted to assess the level of women’s knowledge about obstetric danger signs and to identify clear strategies that could help to strengthen the women’s knowledge about obstetric danger signs.

## Additional files

Additional file 1: Sample MEDLINE Search Strategy and terms. (DOCX 12 kb)

Additional file 2: Factors affecting women’s knowledge. (DOCX 16 kb)

Additional file 3: Quality assessment of articles (NOS for cross sectional study). (DOCX 13 kb)

## Abbreviations

ANC: Antenatal care
CINAHL: Cumulative Index to Nursing and Allied Health Literature
EmOC: Emergency obstetric care
JBI: Johanna Briggs Institute
KODE: Knowledge of Obstetric Danger signs in Ethiopia
MMR: Maternal mortality ratio
NOS: Newcastle–Ottawa Scale
PNC: Postnatal Care
PRISMA: Preferred Reporting Items for Systematic Reviews and Meta-Analyses
SNNP: Southern Nations, Nationalities and Peoples’
