# Supplementary material for: WOMEN's Knowledge of Obstetric Danger signs in Ethiopia (WOMEN's KODE):a systematic review and meta-analysis
Source: Syst Rev. 2019 Feb 25;8:63. doi: 10.1186/s13643-019-0979-7 (PMC6388496; doi:10.1186/s13643-019-0979-7)
Supplement: Supplementary file 1 — Sample MEDLINE Search Strategy and terms. (DOCX 12 kb) [file 13643_2019_979_MOESM1_ESM.docx]

**Additional file 1:** Sample MEDLINE Search Strategy and terms

| **#** | **Searches** | **Results** |
| --- | --- | --- |
| 1 | Knowledge.mp. or Knowledge/ | 626832 |
| 2 | Awareness.mp. or Awareness/ | 126925 |
| 3 | understand*.mp. | 921294 |
| 4 | information.mp. | 1094133 |
| 5 | pregnancy danger sign*.mp. or Pregnancy Complications/ | 86447 |
| 6 | Obstetric Labor Complications/ or obstetric danger sign*.mp. | 16765 |
| 7 | Obstetric complication*.mp. | 3168 |
| 8 | pregnancy warning sign*.mp. | 1 |
| 9 | Obstetric warning sign*.mp. | 0 |
| 10 | Gestational danger sign*.mp. | 0 |
| 11 | severe vaginal bleeding.mp. | 56 |
| 12 | Blurred vision.mp. | 3227 |
| 13 | Swelling of face.mp. | 33 |
| 14 | Seizures/ or Convulsion*.mp. | 59861 |
| 15 | prolonged labo?r.mp. or Dystocia/ | 4385 |
| 16 | retained placenta.mp. or Placenta, Retained/ | 1359 |
| 17 | foul smelling vaginal discharge.mp. | 40 |
| 18 | severe lower abdominal pain.mp. | 62 |
| 19 | high grade fever.mp. | 777 |
| 20 | EmOC.mp. | 233 |
| 21 | emergency obstetric complication*.mp. | 8 |
| 22 | Ethiopia.mp. or Ethiopia/ | 12702 |
| 23 | 1 or 2 or 3 or 4 | 2444048 |
| 24 | 5 or 6 or 7 or 8 or 9 or 10 or 11 or 12 or 13 or 14 or 15 or 16 or 17 or 18 or 19 or 20 or 21 | 169885 |
| 25 | 22 and 23 and 24 | 60 |
