# Supplementary material for: WOMEN's Knowledge of Obstetric Danger signs in Ethiopia (WOMEN's KODE):a systematic review and meta-analysis
Source: Syst Rev. 2019 Feb 25;8:63. doi: 10.1186/s13643-019-0979-7 (PMC6388496; doi:10.1186/s13643-019-0979-7)
Supplement: Supplementary file 2 — Factors affecting women’s knowledge. (DOCX 16 kb) [file 13643_2019_979_MOESM2_ESM.docx]

**Additional file 2: Factors affecting women’s knowledge**

1. **Qualitatively**

| Category | Reported Factors | Studies |
| --- | --- | --- |
|  | Younger age | Workineh (2015), {Hibstu, 2017 #180}Hibstu (2017) |
| Socio-demographic | Older age | Tsegaye (2017), Abiyot (2015), Maseresha (2016) |
|  | Maternal education | Bililing (2017), Tsegaye (2017), Hailu (2010), Workina (2014), Hibstu (2017), Solomon(2015), Damme (2016), Endalemaw(2014), Hailu (2014) |
|  | Household income | Workina (2014), Bogale (2015) |
|  | Partner education | Abiyot (2015), Bogale (2015), Hibstu (2017) |
|  | Maternal occupation | Bililing (2017), Abiyot (2015), Bogale (2015), Damme (2016), Endalemaw (2014) |
|  | Partner occupation | Hibstu (2017) |
|  | Marital status | Hailu (2010) |
|  | Urban residence, | Hailu (2010), Hibstu (2017), Solomon(2015), Maseresha (2016), Bogale (2015) |
| Reproductive history | Higher Gravidity and parity | Hailu (2010), Abiyot (2015), Maseresha (2016) |
| Health service use | ANC utilization | Solomon(2015), Maseresha (2016), Endalemaw (2014), Bogale (2015), Bililing (2017) |
|  | Previousely Gave birth at health facility | Hailu (2014), Bililing (2017), Tsegaye (2017) |
|  | Satisfaction to service | Tsegaye (2017) |
|  | Distance < 30 minutes from facility | Hibstu (2017), Bogale (2015) |
| Misceleneous | Exposure to media | Damme (2016), Hailu (2014), |
|  | Autonomy | Tsegaye (2017), Workineh (2015), |

1. **Quantitatively**

| Authors and year | Region | Recruitment year | Setting | Spontaneous response | Study design | Respondents | **Women’s Knowledge of obstetric danger signs during** | | |
| --- | --- | --- | --- | --- | --- | --- | --- | --- | --- |
|  |  |  |  |  |  |  | **Pregnancy n (%)** | **Childbirth** **n (%)** | **Postpartum n (%)** |
| Abiyot T et al (2015) | Tigray | 2013 | Facility | At least 2 | Cross sectional | 359 | 296 (82.5) | - | - |
| Bililign N. et al (2017) | Amhara | 2016 | Community | At least 3 | Cross sectional | 493 | 230 (46.7) | 137 (27.8) | 130 (26.4) |
| Bogale D. et al (2015) | Oromia | 2013 | Community | At least 3 | Cross sectional | 562 | 179 (31.9) | 152 (27) | 124 (22.1) |
| Damme T. G. et al (2016) | Oromia | 2015 | Facility | At least 3 | Cross sectional | 198 | 152 (76.8) | 154 (77.8) | 129 (65.5) |
| Endalemaw M. et al (2014) | Amhara | 2012 | Facility | At least 3 | Cross sectional | 385 | 181(47) | 176(45.7) | - |
| Hailu D. et al (2014) | Tigray | 2013 | Community | At least 2 | Cross sectional | 485 | 285 (58.8) | 299 (61.6) | - |
| Hailu M. et al (2010) | SNNPR | 2007 | Community | At least 2 | Cross sectional | 743 | 226 (30.4) | 305 (41.3) | 279 (37.7) |
| Hibstu D. T. et al (2017) | SNNPR | 2016 | Facility | At least 3 | Cross sectional | 342 | 168 (49.1) | 181 (52.9) | 153 (44.7) |
| Maseresha N et al (2016) | Somali | 2014 | Community | At least 2 | Cross sectional | 632 | 201 (31.8 ) | 161 (25.5) | 121(19.1) |
| Workineh Y. et al (2014) | SNNPR | 2014 | Community | At least 2 | Cross sectional | 390 | 184 (47.2) | 193(49.5) | 285(73) |
| Solomon A. et al (2015) | Amhara | 2014 | Facility | At least 2 | Cross sectional | 355 | 137(38.6) | - | - |
| Tsegaye D. et al (2017) | Oromia | 2015 | Facility | At least 2 | Cross sectional | 831 | 309 (37.3) | 194 (23.3) | 30 (3.6) |
