# Supplementary material for: WOMEN's Knowledge of Obstetric Danger signs in Ethiopia (WOMEN's KODE):a systematic review and meta-analysis
Source: Syst Rev. 2019 Feb 25;8:63. doi: 10.1186/s13643-019-0979-7 (PMC6388496; doi:10.1186/s13643-019-0979-7)
Supplement: Supplementary file 3 — Quality assessment of articles (NOS for cross sectional study). (DOCX 13 kb) [file 13643_2019_979_MOESM3_ESM.docx]

Quality assessment of articles (NOS for cross sectional study)

| Studies | Selection | | | | Comparability | Outcome | | Total score |
| --- | --- | --- | --- | --- | --- | --- | --- | --- |
|  | Representativeness  (1) | Sample size  (1) | Non-respondents  (1) | Ascertainment of the exposure (risk factor)  (2) | The subjects in different outcome groups are comparable, based on the study design or analysis. Confounding factors are controlled (2) | Assessment of the outcome  (2) | Statistical test  (1) |  |
| Abiyot T et al. (2015) | * | * | * | ** | * | * | * | 8 |
| Bililign N et al. (2017) | * | * | * | ** | ** | * | * | 9 |
| Bogale D et al. (2015) | * | * | * | ** | * | * | * | 8 |
| Damme T G (2016) | * | * | * | ** | * | * | * | 8 |
| Mengesha E et al. (2015) | * |  | * | ** | * | * | * | 7 |
| Hailu D et al. (2014) | * | * | * | ** | * | * | * | 8 |
| Hailu M et al. (2010) | * | * | * | ** | * | * | * | 8 |
| Hibstu DT et al. (2017) | * | * | * | ** | ** | * | * | 9 |
| Maseresha N et al. (2016) | * | * | * | ** | ** | * | * | 9 |
| Workineh Y et al. (2014) | * | * | * | ** | * | * | * | 8 |
| Solomon AA et al. (2015) | * | * | * | ** | * | * | * | 8 |
| Tsegaye D. et al (2017) | * | * | * | ** | * | * | * | 8 |
